# A Combined Targeted and Whole Exome Sequencing Approach Identified Novel Candidate Genes Involved in Heritable Pulmonary Arterial Hypertension

**DOI:** 10.1038/s41598-018-37277-0

**Published:** 2019-01-24

**Authors:** Chiara Barozzi, Margherita Galletti, Luciana Tomasi, Sara De Fanti, Massimiliano Palazzini, Alessandra Manes, Marco Sazzini, Nazzareno Galiè

**Affiliations:** 1 Department of Experimental, Diagnostic and Specialty Medicine, University of Bologna, Bologna, Italy; 2 Department of Biological, Geological and Environmental Sciences, Laboratory of Molecular Anthropology & Centre for Genome Biology, University of Bologna, Bologna, Italy; 3 National Institute of Biostructures and Biosystems (NIBB), Rome, Italy

## Abstract

The pathogenesis of idiopathic and heritable forms of pulmonary arterial hypertension is still not completely understood, even though several causative genes have been proposed, so that a third of patients remains genetically unresolved. Here we applied a multistep approach to extend identification of the genetic bases of such a disease by searching for novel candidate genes/pathways. Twenty-eight patients belonging to 18 families were screened for *BMPR2* mutations and *BMPR2*-negative samples were tested for 12 additional candidate genes by means of a specific massive parallel sequencing-based assay. Finally, whole exome sequencing was performed on four patients showing no mutations at known disease genes, as well as on their unaffected parents. In addition to *EIF2AK4*, which has been already suggested to be associated with pulmonary veno-occlusive disease, we identified the novel candidate genes *ATP13A3*, *CD248*, *EFCAB4B*, involved in lung vascular remodeling that represent reliable drivers contributing to the disease according to their biological functions/inheritance patterns. Therefore, our results suggest that combining gene panel and whole exome sequencing provides new insights useful for the genetic diagnosis of familial and idiopathic pulmonary arterial hypertension, as well as for the identification of biological pathways that will be potentially targeted by new therapeutic strategies.

## Introduction

Pulmonary arterial hypertension (PAH) is a rare and severe disease affecting small pulmonary arteries and caused by abnormal proliferation of their smooth muscle cells and endothelial cells leading to an increase in pulmonary vascular resistance and right ventricular failure^1,2^. Despite appreciable improvements in the treatment of such a pathological condition, PAH remains a fatal disease characterized by high mortality.

To date, the pathogenesis of both idiopathic and heritable forms of PAH (IPAH and HPAH) is still not completely understood. However, a number of potentially causative mutations in genes primarily related to PAH have been discovered via conventional linkage analyses and, more recently, thanks to massive parallel sequencing (or next-generation sequencing, NGS) approaches. In particular, the first reports of genetic contributions to PAH were identified by linkage studies in which mutations in the gene encoding the bone morphogenetic protein receptor type 2 (*BMPR2*) were found in approximately 75% of cases of HPAH and in about 20% of patients with IPAH^3–6^. Later, mutations in genes related to the transforming-growth factor β signalling (*ALK1*, also called *ACVRL1*, and *ENG*), which are known to contribute to hereditary haemorrhagic telangiectasia, were reported to be associated also with PAH^7,8^. Moreover, the same association pattern was observed for mutations in SMADs, which are involved in the transforming-growth factor β/BMP signalling^9,10^.

In the last few years, the ever-decreasing per-base costs achieved by NGS technologies and the development of more powerful statistical and bioinformatics tools enabled the design of innovative studies based on whole exome-sequencing (WES) or whole-genome sequencing (WGS) data. This led to the rapid identification of many rare variants, some with a large effect size, thus considerably improving the understanding of the genetic determinants of both Mendelian and complex traits^11^. Interestingly, application of WES to the study of PAH successfully pointed out novel mutations in the gene encoding caveolin 1 (*CAV1*)^12^ and in *KCNK3* in patients affected by HPAH or IPAH^13^. Another WES-based study linked recessive variants of a single gene, *EIF2AK4*, to pulmonary veno-occlusive disease development in 13 PAH families^14^. More recently, by analyzing WES data from 12 unrelated patients with IPAH, but lacking *BMPR2* mutations, the DNA topoisomerase II-binding protein 1 (*TOPBP1*) gene, which is involved in the response to DNA damage and replication stress, was identified as a risk factor for IPAH^15^.

According to these promising findings, genetic assessment of PAH might play a more prominent role within current and future diagnostic algorithms, as also indicated in the new European Society of Cardiology/European Respiratory Society (ESC/ERS) guidelines and may even be useful for risk stratification^2^. Today, PAH genetic analyses are focused on the sequencing of coding regions of the *BMPR2*, *ALK1*, *ENG*, *SMAD9*, *CAV1*, *KCNK3* and *EIF2AK4* genes. Nevertheless, defects in the genes belonging to the *BMPR2*/*Alk1*-signalling pathway or in other loci plausibly involved in the pathogenesis of PAH are not able to elucidate the full spectrum of HPAH manifestations.

That being so, in the present study we applied the most up-to-date massive parallel sequencing techniques to develop a combined targeted- and trio-WES-based approach aimed at providing new insights into the genetic bases of HPAH by searching for novel disease genes in patients harbouring no mutations in the previously described candidate genes. In fact, with respect to proband-WES, trio-WES has been proved to ensure a superior molecular diagnostic rate (i.e. 31% in trio-WES [95%CI, 27–36%] compared to 22% in proband-WES [95%CI, 18–27%], *P* = 0.003)^16^.

**Figure 1:**
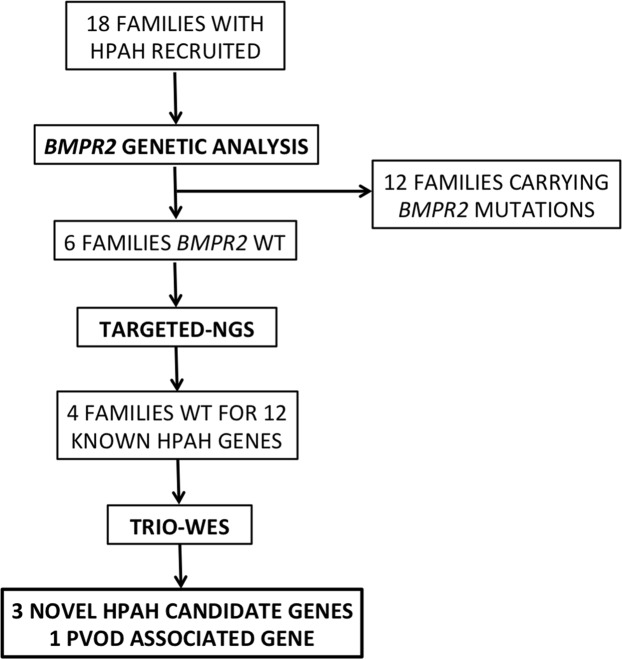
Flowchart outlining the experimental design of the study, the criteria used for selecting the examined patients and the results achieved by each step. HPAH, heritable pulmonary arterial hypertension; WT, wild-type; TARGETED-NGS, specific massive parallel sequencing protocol developed to test 12 known HPAH candidate genes at once; TRIO-WES, whole-exome sequencing of trios including HPAH patients and their unaffected parents; PVOD, pulmonary veno-occlusive disease.

For this purpose, 28 HPAH patients belonging to 18 different families were screened for *BMPR2* mutations and/or rearrangements in order to identify *BMPR2*-negative samples to be tested for 12 additional candidate genes thanks to the development of a specific NGS panel. Results obtained by these preliminary evaluations then drove the selection of the most informative HPAH probands to be sequenced for the whole exome together with their unaffected parents. A flowchart outlining the described study design, as well as samples examined and results achieved is depicted in Fig. 1.

Such an experimental design thus promised to maximize the chance to detect novel genetic variants and altered functional pathways potentially contributing to the complex etiology of HPAH.

## Results

### *BMPR2* Screening of the Whole HPAH Cohort

Pedigrees and main clinical parameters of patients included in the study are reported in Supplementary Figs S1 and S2 and in Supplementary Table S1, respectively.

Among the 28 HPAH subjects examined, *BMPR2* mutations were detected in 19 patients (68%), accounting for 12 out of the 18 investigated families (Table 1).

**Table 1:** BMPR2 mutations in 12 Italian families with HPAH.

Fam	Exon	Nucleotide change	Amino acid change	Study	SIFT	PPH2	MA	SNP&go	MutPred	Condel
1	1	−155 insTCGGTCCT 21 delGCCCTGGC 29 G/A	p.P8fsX27	Machado *et al*.^1^						
2	3	352 T > A	p.C118S	This study	0.01	1	2.92	0.82	0.888	0.747
3	6	631 C > T	p.R211X	Thomson *et al*.^2^						
4	7	935 T > G	p.L312R	This study	0	0.99	4.27	0.83	0.952	0.99
5	8–9	Del exons 8–9	p.?	Aldred *et al*.^3^						
6	Intr9	1276 + 4 A > G	Ex 9 skipped	Machado *et al*.^1^						
7	9	1181 T > C	p.L394S	This study	0.01	1	1.72	0.80	0.511	1
8	9	1246 insG	p.I416fs	Machado *et al*.^1^						
9	Intr10	1414-2 A > G	p.?	This study						
10–11	12	2695 C > T	p.R899X	Lane *et al*.^4^						
12	12	2789 C > G	p.S930X	Morisaki *et al*.^5^						

Fam, family. Functional damaging effect was predicted according to the following scores: SIFT <0.05; PPH2, Polyphen2 >0.5; MA, Mutation Assessor >0.65; SNP&go >0.5; MutPred g_score >0.5, actionable hypothesis; g_score >0.75, confident hypothesis; Condel >0.52. Studies previously describing the considered variant were reported in Supplementary Information.

In detail, we described 10 single nucleotide variants (SNVs) (i.e. three missense, four nonsense, one frame-shift, and two splice-site variants), which have been previously reported as pathogenic in literature^17^, or predicted as potentially disease-associated by *in silico* approaches. Moreover, we detected a short insertion/deletion (INDEL) in exon 1 and a deletion spanning exons 8 and 9.

### Evaluation of *BMPR2*-negative Samples via Targeted NGS

Nine patients belonging to six different families showed no *BMPR2* mutations or structural rearrangements. Six of them (i.e. one per family) were thus characterized for 12 additional candidate genes thanks to the design and implementation of a specific NGS panel. The total amount of sequence reads mapped on the targeted genomic regions was of 3,090,821 (i.e. approximately 97% of those generated by the performed multiplex sequencing experiment), with on average 502,900 reads per sample and resulting in a mean coverage of 2,305X per sample (mean uniformity 95.25%). Synonymous variants were not taken into further consideration, while functional predictions were performed for the eight intronic variants detected using the Human Splicing Finder 3.0 tool, which pointed out no significant alterations (i.e. HSF score > 80) of splicing sites or enhancer regions.

**Figure 2:**
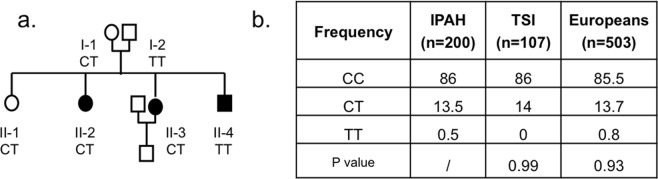
Segregation pattern of *TopBP1* rs55633281 and its allele frequency in validation cohorts. (**a**) Segregation pattern of *TopBP1* rs55633281 in the whole family of trio 4. Index patient II-2. Symbol key: circle, female; square, male; solid symbols, PAH diagnosis. (**b**) Allele frequency of *TopBP1* rs55633281 in the cohort of IPAH patients (n = 200), TSI (n = 107) and European (n = 503) 1000 Genome Project populations. No differences were found according to chi-square test (p > 0.05). Plots were created using the R software v.3.3.2 (R: A Language and Environment for Statistical Computing, R Core Team, R Foundation for Statistical Computing, Vienna, Austria (2016) https://www.R-project.org).

Interestingly, we identified non-synonymous variants only on the *TopBP1* gene: three previously reported in IPAH by Perez *et al*.^15^ (rs55633281, rs17301766 and rs10935070) and rs3192149. For these latter variants genotype frequencies in our patient cohort did not differ from those expected in the general European population (Fisher test, p > 0.05) and, in particular, in the sole Italian population sample sequenced by the 1000 Genomes Project (i.e. Tuscans from Italy, TSI). As regards rs55633281, it was suggested to likely increase susceptibility to small vessel loss in PAH^15^. Accordingly, we analyzed its segregation in all the affected and unaffected relatives of the proband carrying the single nucleotide polymorphism (SNP), but no cosegregation between the variant allele and the disease was found (Fig. 2a). Furthermore, we evaluated rs55633281 incidence in 200 additional IPAH patients, but we found no differences between the genotype frequencies observed in the disease cohort and those reported for the European and TSI 1000 Genomes Project populations, as evaluated using Chi square test (Fig. 2b).

### Whole Exome Sequencing of Trios Showing No Mutated Candidate Genes

Subsequent to the screening of the mutational profiles of *BMPR2* and of the additional PAH candidate genes included in the developed NGS panel, four patients showing no sequence variants and/or chromosomal rearrangements in the investigated genomic regions were submitted to trio-WES experiments together with their unaffected parents.

We thus generated high coverage exome data (on average 190–200X per sample) overall showing more than 96% and 90% of the targeted nucleotide positions covered respectively by at least 10 and 20 reads after application of filtering and fragments de-duplication procedures (Table 2). This sequence depth enabled us to identify approximately 116,000–125,000 variants per sample, which were submitted to stringent quality control and filtering procedures (see Materials and Methods).

**Table 2:** Summary of overall output and filtered variants for the performed trio-Whole exome sequencing experiments.

Trio	% 10X reads	% 20X reads	Number of variants	LoF/Missense	Compound heterozygous	Homozygous variants	De novo variants
Proband 1	96.55	91.72	116,189	858	33 (14 genes)	3	6
Mother	96.86	93.01	117,082				
Father	97.37	91.84	115,855				
Proband 2	96.78	92.52	123,862	1005	48 (20 genes)	9	12
Mother	97.21	91.60	123,725				
Father	98.37	95.71	123,111				
Proband 3	96.34	86.23	121,215	847	54 (23 genes)	16	8
Mother	97.65	93.36	121,163				
Father	97.45	92.79	122,161				
Proband 4	97.37	93.12	125,668	885	70 (23 genes)	5	3
Mother	97.24	93.95	126,700				
Father	97.26	92.61	124,454				

% 10X reads, percentage of nucleotide positions covered by at least 10 reads after application of filtering and fragments de-duplication procedures; % 20X reads, percentage of nucleotide positions covered by at least 20 reads after application of filtering and fragments de-duplication procedures; LoF, Loss of Function.

In particular, attention was focused on heterozygous variants showing alternate allele frequency lower than 5% in the combined reference populations of European ancestry (i.e. ExAC/1000Genomes/ESP6500), including compound heterozygous sites, as well as on homozygous changes (Table 2). Conversely, due to the disease transmission pattern observed in the four examined families, *de novo* variants were not considered in subsequent analyses. Moreover, in order to validate the obtained results, 26 of the detected variants (about 10%) were confirmed by Sanger sequencing. In particular, 16 of them were chosen according to the applied prioritization filtering procedures (i.e. 12 compound heterozygous and three homozygous variants), while ten were randomly selected (Supplementary Table S2).

In trio 1, only variants at two genes (i.e. *ADAT* and *ATP13A3*) passed the applied filtering procedures and especially the latter gene was considered as a candidate HPAH-associated locus according to both biological significance and specific expression in diseased tissue (i.e. lung, smooth muscle cells and cardiomyocytes). Moreover, one currently unaffected sibling of the proband was tested for this variant resulting heterozygous as the parents.

Four mutated genes observed in trio 2 passed the filtering procedure and are reported here in order of prioritization by biological function: *EFCAB4B*, *VPS18*, *LRRC40* and *PHKB*. The latter was discarded because of its association with glycogenosis, which was absent in the proband.

Interestingly, among the 16 biallelic variants observed in the proband of trio 3, we identified a damaging nucleotide substitution in the Eukaryotic translation initiation factor 2 alpha kinase 4, *EIF2AK4* (NM_001013703.3, c.1387 C > T), which leads to a loss of function mutation (Arg463Ter). This gene has been already found to be associated to pulmonary veno-occlusive disease (PVOD)^14^.

As regards trio 4, we evaluated variants segregation of six candidate genes (i.e. *ZP4, RABEPK, ACAD10, DNAH7, KCNT1* and *CD248*) resulting from the previously described filtering analysis in the remaining affected and unaffected members of the family. Among these loci, only *CD248* showed a segregation pattern compatible with a potential involvement in HPAH. Moreover, the observed NM_020404.2, c.683 C > T substitution leads to a Pro228Leu change that was predicted as damaging by all the used *in silico* tools. Details of the prioritized variants are summarized in Table 3 and additional information are reported in Supplementary Table S3.

**Table 3:** Autosomal recessive and compound heterozygous variants detected with trio-Whole exome sequencing.

Trio	Gene	Chromosome Position	Ref/Alt	Effect	Inherited	dbSNP	Freq	Damaging
Trio 1	*ATP13A3*	3:194151733	C/T	M	AR		0	5 of 6
	*ADAT1*	16:75642801	G/A	M	AR	rs77029992	0.0189	5 of 6
Trio 2	*EFCAB4B*	12:3768803	T/G	M	AR	rs201641139	0	4 of 6
	*VPS18*	15:41192187	G/A	M	AR	rs202222195	0	5 of 6
	*EP400*	12:132505812	A/G	M	Father		0	4 of 6
	*EP400*	12:132547094	−/CAGCAG	IN	Mother	rs528214697	0	
	*LRRC40*	1:70614325	−/A	FR	Father	rs763171257	0	
	*LRRC40*	1:70625071	G/A	M	Mother	rs145682711	0.001	4 of 6
	*PHKB*	16:47536996	G/A	M	Father	rs144486825	0.001	5 of 6
	*PHKB*	16:47549473	G/T	M	Mother	rs56257827	0.0119	6 of 6
	*PHKB*	16:47549492	A/G	M	Mother	rs117218785	0.002	4 of 6
Trio 4	*CD248*	11:66083816	G/A	M	AR	rs149949198	0.004	6 of 6
	*KCNT1*	9:138664772	C/G	M	Father	rs148162797	0	4 of 6
	*KCNT1*	9:138678332	G/A	M	Mother	rs867696317	0	5 of 6
	*OBSCN*	1:228539083	C/T	M	Mother	rs754776763	0	5 of 6
	*OBSCN*	1:228556519	A/T	M	Father		0	5 of 6
	*RABEPK*	9:127982839	C/A	M	Mother	rs74769898	0.003	5 of 6
	*RABEPK*	9:127982852	C/−	FR	Father	rs546948946	0.004	
	*ACAD10*	12:112159522	T/C	M	Father		0	5 of 6
	*ACAD10*	12:112186274	C/T	M	Mother	rs34245489	0.0418	4 of 6
	*DNAH7*	2:196602773	G/A	M	Father	rs114621989	0.0109	5 of 6
	*DNAH7*	2:196726484	C/T	M	Mother	rs201185180	0.002	5 of 6
	*ZP4*	1:238048530	G/A	M	Mother	rs139132490	0	6 of 6
	*ZP4*	1:238050081	T/C	M	Father	rs36017138	0.007	6 of 6

Data were filtered for frequency <5% and for functional impact prediction. Freq, allele frequency in the 1000 Genomes Project phase 3 dataset; Damaging, only mutations suggested as potentially damaging by at least four out of six bioinformatic tools (dbNSFP Functional Predictions and Scores 3.0) were shown. Candidate genes proposed by the present study are underlined. M, missense variant; IN, in-frame insertion; FR, frameshift variant; AR, autosomal recessive.

Finally, enrichment of specific Gene Ontology (GO) terms in the genomic regions affected by SNVs and INDELs having passed stringent filtering procedures was tested to identify the most relevant functional pathways plausibly involved in the development of HPAH (Supplementary Table S4). Accordingly, in the proband belonging to trio 1 an enrichment was observed only for genes playing a role in ATPase activity coupled to transmembrane movement of substances (GO:0042626). Significantly enriched GO terms among the genes harboring variants in the proband belonging to trio 2 turned out to be associated to biological processes involved in ectoderm development, cell-cell adhesion, and cellular component movement (GO:0007398, GO:0016337, GO:0006928). In particular, loci belonging to the cadherin protein class, as well as genes involved in the cadherin and in the Wnt signaling pathways resulted overrepresented in the obtained gene list with respect to the background set of known human genes. Significant overrepresentation of loci involved in the 5-Hydroxytryptamine degradation pathway was finally found among genes showing variants in the proband belonging to trio 4, coupled with barely significant results pointing to an enrichment of loci exerting a molecular function ascribable to oxidoreductase activity (i.e. acting on the aldehyde or oxo group of donors).

## Discussion

Despite recent advances in the identification of novel candidate genes triggering PAH^12,13^, the full spectrum of the genetic bases underlying such a disease remains widely unexplored. In fact, mutations in *BMPR2* and in other not so frequently altered loci were found to account for less than 80% of heritable PAH cases^18^. In line with these findings, our data confirm that about one third of familial cases remains genetically unresolved. In this scenario, approaches based on powerful and yet cost-effective massive parallel sequencing techniques represent a promising tool to achieve a deeper understanding of the molecular basis of PAH. Genetic evaluation might thus pave new ways for current and future diagnostic algorithms, as envisaged by the ESC/ERS guidelines and will be helpful for developing innovative therapeutic strategies targeting new candidate disease-associated biological pathways in a personalised precision-medicine perspective.

According to this rationale, the multistep approach implemented in the present study combined direct sequencing of *BMPR2*, targeted NGS of a gene panel designed to include all PAH candidates identified so far, and WES of patients showing no mutations at known disease genes together with their unaffected parents. This strategy allowed us to refine the selection of samples to be submitted to WES and hence to maximize cost-effectiveness, as well as to simplify data analysis, which represents the major challenge when dealing with genome-wide or exome-wide experimental approaches. We therefore selected a subset of patients that maximized the chance of identifying novel genes responsible for PAH. In fact, although this strategy reduces the number of patients meeting the consecutive inclusion criteria, thus limiting this approach only to large referring centers able to enroll wide patients cohorts, by performing trio-WES we succeeded in pinpointing only genetic variants following the predicted transmission pattern and therefore in increasing the likelihood of successful diagnosis^19^.

For this purpose, analyses of WES data were performed by applying a particularly stringent filtering procedure based on functional evaluation of the detected variants using several *in silico* tools. Combining multiple predictions has indeed the potential to improve the recognition of disease-associated mutations by considering them with different weight according to evolutionary conservation metrics and estimates of the biochemical consequence of the related amino acid changes^20^. Moreover, our analysis has been further supported by the use of data from a cohort of supercentenarians, which were used as reference to filter for rare variants not to be considered as damaging.

Interestingly, among the genes passing the applied filtering procedure, we identified two PAH-associated candidates in two different subjects, both being involved in cation transport: *ATP13A3*, a membrane ATPase belonging to the P5 family, and *EFCAB4B*, EF-hand Calcium binding domain containing protein 4B. As recently reviewed by Olschewski *et al*.^21^, the contribution of alterations at ion channels to pulmonary hypertension is progressively emerging because they are supposed to play a pivotal role in both the triggering and the development of the disease, as demonstrated by the function of *KCNA5* and *KCNK3* in regulation of pulmonary vascular tone. Potassium transport through these channels is known to contribute to the maintenance of resting membrane potential in smooth muscle cells of pulmonary arteries (PASMCs), which regulates cytoplasmic calcium levels by voltage-sensitive Ca^2+^ channels. In fact, inhibition of K^+^ channels induces membrane depolarization leading to acute vasoconstriction. Furthermore, it has recently demonstrated that reduced K^+^ channel activity in PASMCs promotes cell proliferation and prevents apoptosis, thus contributing to vascular remodeling^22^. In particular, mutations in *KCNK3* are well-established causes of idiopathic (1.3%) and heritable (3.2%) PAH characterized by dominant transmission pattern with incomplete penetrance.

In detail, according to data available in the Human Protein Atlas and GTEx databases we prioritized *ATP13A3* based on its expression in lung, cardiomyocytes and smooth muscle cells, which are involved in pulmonary hypertension vascular remodeling. Although its function remains currently widely unknown, it is a cation channel expressed at high rate in smooth muscle cells, which represent the main player in vascular tone regulation. Our data thus suggest that it could likely contribute to the initiation of PAH similar to *KCNK3* and *KCNA5*. In line with these findings, in a very recent paper having analyzed 1,038 whole genome sequences from idiopathic and familial PAH, heterozygous mutations at *ATP13A3* showing functional impact on protein catalytic activity were observed in 11 patients^23^. Moreover, *ATP13A3* mRNA expression was confirmed in primary PASMCs and endothelial cells where its loss hindered proliferation and enhanced apoptosis of endothelial cells, which is known as the initiation event of PAH^23^. The role of *EFCAB4B* (or *CRACR2A*) was instead firstly highlighted in immune cells^24^ regulating store-operated calcium entry (SOCE). Increased SOCE has been then associated to PASMCs proliferation, migration and atypical vascular remodeling^25,26^. Such an EF-hand containing protein interacts directly with other critical components of SOCE channels (i.e. Orai1 and STIM1) forming a tertiary complex able to turn on Calcium influx into the endoplasmic reticulum. *EFCAB4B* transcripts are expressed at high levels in lung^24^ and we suggest that any disruptive variants at this gene could interfere with Calcium homeostasis of smooth muscle cells in which intracellular levels are finely regulated to control vascular tone.

Although the described findings are in agreement with the long established importance of ion channels (K^+^ and Ca^2+^ in particular) in the development of PAH^22,27^, the trio-WES approach implemented in the present study provided insights also on genes involved in processes leading to pulmonary vasculature growth and remodeling, but previously not identified as candidate disease-associated loci. Among them, the transmembrane glycoprotein CD248, also known as endosialin or Tumour endothelial marker 1 (*TEM1*), was found to be mutated in homozygosis in all the affected siblings of proband 4, whereas the unaffected sister and parents are proved to be heterozygous for the same variant. Moreover, all the used predicting tools recognized the related amino acid substitution as damaging. Interestingly, CD248 is a member of the C-type lectin receptor superfamily originally identified in tumor blood vessels^28^. In fact, it is known to be expressed during embryogenesis, but almost completely inactivated after birth except in a minority of cells, such as a subset of perivascular cells in lungs^29^. On the other hand, it is reactivated in nearly all tumor types, as well as in some inflammatory diseases (e.g. arthritis^30^). Using a monoclonal antibody directed to CD248, Rybinski and colleagues showed that its reduced expression on pericytes was accompanied by alteration in α-SMA expression pattern, morphological changes and depolarization of both pericytes and endothelial cells during angiogenesis, leading finally to the formation of dysfunctional microvessels^31^. Reduced pericytes proliferation and migration, as well as weakened interactions between pericytes and endothelial cells, were previously reported in PAH leading to impaired regeneration of functional vessels and loss of peripheral pulmonary arteries, which are key pathological features of the disease^32^. Coupled with our results, these findings suggest a pivotal role of CD248 in altered remodeling of lung blood vessels, which could eventually lead to PAH. CD248 is also known to contribute to the intracellular signal of tyrosine kinase receptors, such as *PDGFR*^33^ and *TGF-β R*^34^, although its function remains unknown. Further studies will be thus required to specifically investigate interactions between CD248 and BMPR2 in lungs.

Finally, one patient was found to harbor a pathogenetic mutation in *EIF2AK4*, a gene already associated to PVOD^14^, an uncommon form of pulmonary hypertension with a preferential involvement of the pulmonary post-capillary system. *EIF2AK4* mutations were also detected in a large cohort of patients with clinical diagnosis of PAH^35^. Because of the complexity of differential diagnosis between PAH and PVOD due to similarity in radiological and histological features, genetic analyses will be thus of considerable support to avoid misclassification of such patients. Actually, the several pharmacological strategies available for PAH are ineffective or even harmful on PVOD patients, therefore an appropriate classification by means of genetic testing appears to be mandatory for risk stratification and management of these patients.

## Conclusions

While in some instances NGS may represent an important tool to sustain clinical diagnosis, as is the case represented by the detection of *EIF2AK4* biallelic mutations in patients with PAH, in most cases these analyses require detailed biological confirmations, in absence of literature reports. Our stringent filtering and prioritization workflow instead succeeded in pinpointing genes (e.g. *ATP13A3*) that are still poorly elucidated from both molecular and pathogenetic perspectives, but which promise to be reliable candidates contributing to the development of PAH according to their biological functions and inheritance patterns. Our data were not conclusive about the exact role of these potentially disease-loci, but lay the foundation for future functional investigations specifically aimed at addressing this issue.

We therefore propose a comprehensive targeted-NGS panel for a molecular diagnosis of familial and idiopathic PAH patients aimed at broadening the range of genetic analyses currently implemented in such a research field. For this purpose, in addition to the known PAH-associated genes, we suggest to consider also the three candidate loci here discovered via trio-WES (i.e. *ATP13A3*, *EFCAB4B*, *CD248*). Moreover, it was recently suggested by Perez *et al*. that also *TOPBP1* could act as a modifier gene in the development of PAH^15^ and it was thus included in subsequent PAH-specific NGS panels^36^. However, our results on segregation patterns of *TOPBP1* variants in a family of affected carriers, coupled with extensive analysis of its most replicated candidate SNP rs55633281 on 200 PAH patients, revealed no differential occurrence of this nucleotide substitution between disease and healthy subjects. Accordingly, we suggest to exclude this gene from further NGS evaluations, at least as concerns the analysis of patients of Italian and, more in general, European ancestry.

The new PAH-specific gene panel based on the candidate genes proposed is thus expected to increase the probability to detect disease-causing mutations and consequently to reduce costs, time, and research effort per patient. Moreover, improving diagnosis can overcome adverse effects experienced by patients and their relatives, such as failure in identifying appropriate treatments, in considering risks related to future pregnancies, as well as in timely providing reliable prognosis. Finally, given the persistent high mortality in PAH and the current inability to predict patients drug responsiveness, a future expansion of the proposed NGS-based pipeline of analysis could be aimed at matching a particular genotype to the most likely effective drug, allowing for tailored approaches to the clinical management of the disease.

## Materials and Methods

### Patients

Twenty-eight consecutive subjects affected by HPAH and belonging to 18 different families were recruited at the main Italian referral centre for Pulmonary Hypertension in Bologna since 2002 and under the approval of the Ethical Committee of the S. Orsola-Malpighi Hospital. Families were identified by the presence of at least two members showing typical clinical manifestations of PAH, as documented by interviews aimed at reconstructing the detailed family histories. Written informed consent was obtained for all participants and the present study was designed and conducted in accordance with relevant guidelines and regulations of the ethical principles for medical research involving human subjects stated by the WMA Declaration of Helsinki.

All patients were of European ancestry and belonged especially to the Italian and the Slovenian populations, which have been demonstrated to share a considerable fraction of their overall genomic background^37^.

### Mutation Analysis of the *BMPR2* Gene

According to the 2015 conjoint ERS and ESC guidelines for the diagnosis and treatment of pulmonary hypertension^2^, genetic screening of *BMPR2* was primarily performed.

DNA was extracted from blood samples by means of an automated DNA Purification System (MagCore HF16, RBC Bioscience Corp., Taiwan). *BMPR2* mutational profiles were first assayed by Denaturing High-Performance Liquid Chromatography (DHPLC) and, in case of differences with respect to the wild-type control, further characterised by Sanger sequencing on an ABI 3730 instrument. Samples showing no mutations were also submitted to the screening of *BMPR2* large rearrangements using the SALSA multiplex ligation-dependent probe amplification (MLPA) P093-C HHT/PPH1 probe mix kit (MRC Holland, Amsterdam, The Netherlands) according to the manufacturer’s instruction.

### Creation of a Targeted NGS Panel

*BMPR2* mutations or structural rearrangements were not detected in subjects belonging to six families so that one patient per family was subsequently analysed for other well-established candidate genes by developing a specific NGS panel comprising: *BMPR2* (including 5′UTR), *BMPR1A*, *BMPR1B*, *ACVRL1*, *ENG* (including 5′UTR), *SMAD1*, *SMAD4*, *SMAD9*, *CAV1* (including 3′UTR), *KCNA5* (including 5′UTR), *KCNK3* and *TopBP1*^4,7–10,12,13,15,38^.

The panel was designed with the *in silico* Ion AmpliSeq Designer tool to simultaneously characterize all the selected genes at once and by considering an exon padding of 25 nucleotides, thus enabling the generation of 190 amplicons with a length ranging between 125 and 375 nucleotides. Barcoded libraries were then prepared using the Ion AmpliSeq Library Kit 2.0 and DNA fragments were clonally amplified onto the Ion Sphere™ particles via emulsion PCR. Massive parallel sequencing of the amplified barcoded libraries was finally performed on an Ion Torrent PGM platform using a 316 v.4 chip (all kits and informatics tools are by Thermo Fisher Scientific, Carlsbad, CA, USA).

Processing of the obtained raw sequence reads and quality controls were performed using tools implemented in the Torrent Suite v.4.4.3. Variants calling and annotation was then performed with the Variant Caller v.4.4.3 and Ion Reporter Software v.4.0, while the Integrative Genome Viewer tool was used to perform coverage analysis, as well as to confirm sequence quality and the identified variants by means of visual inspection.

### Whole Exome Sequencing and Annotation

Four HPAH patients out of the six showing no *BMPR2* mutations/rearrangements and no potentially disease-associated variants at the other candidate genes included in the developed NGS panel were selected for trio-WES experiments.

Samples have been processed with the SeqCap EZ MedExome Target Enrichment Kit (Roche Sequencing, Pleasanton, CA, USA) and sequenced using the Nextseq500 platform (Illumina, San Diego, CA, USA) available at the Personal Genomics facilities (Verona, Italy) to obtain an average coverage of 190-200X per sample. Sequenced reads were mapped to the hg19 reference genome using iSAAC align version 03.16.12.05. The iSAAC Variant Caller version 1.0.7 was finally used to identify SNVs and INDELs.

Clinically significant variants were annotated using the Variant Annotation Tool^39^, which contains annotation information from the following resources: RefSeq Genes 105v2, NCBI; dbSNP v.149, NCBI; ClinVar 2017-01-06, NCBI; HGMD; OMIM Genes 2010-10-27; ExAC Variant Frequencies v.0.3, BROAD; NHLBI ESP6500Sl-V2-SSA137 Exome Variant Frequencies 0.0.30, GHI; 1000Genomes Phase3 Variant Frequencies 5b, dbNSFP Functional Predictions and Scores 3.0, GHI. To refine variants annotation the CentoMD v.3.1-06102016 and the Personal Genomics Variant Database (PGVD) v.1.0.25 were used. In particular, the PGVD is an internal repository of genetic markers that contains a peer-documented combination of all identified variants/mutations associated with phenotype and clinical data. It also includes rare variants observed in a cohort of supercentenarians.

### Variants Prioritization and Filtering Workflow

All the variants considered in the present study were required to satisfy the following quality filters: (1) at any genomic position where a *de novo* variant was called, reads depth should be ≥10 in all the three members of the trio, with both parents showing less than 5% of reads supporting the variant allele; (2) at any genomic position showing recessive genotypes, reads depth should be ≥10 in the proband and in each parent; (3) at any genomic position where a heterozygous variant was called, a threshold of ≥25% of the reads should support the alternative allele.

Variants were further filtered for: (4) their impact on protein structure and function as estimated by at least four out of the six predictive tools used to inform the dbNSFP database of the Variant Annotation Tool^39^; (5) a frequency < 5% in the combined 1000Genomes/ExAc and ESP6500 databases and in the above-mentioned centenarians-based repository. Variants filtered by means of these criteria were finally matched with the Pulmonary Arterial Hypertension Knowledgebase (PAHKB)^40^ and prioritized according to their biological function and relevance in lung and heart tissues by envisioning the most up-to-date literature.

Enrichment of specific GO terms among the genomic regions affected by the SNVs and/or INDELs having passed such stringent filtering steps was also tested using the PANTHER tool^41^ to identify the most relevant functional pathways plausibly involved in the development of HPAH. For each proband, the list of top candidate genes generated through the previously described filtering procedures was compared to the background set of all genes annotated for the human genome and obtained p-values were corrected for multiple testing according to the Bonferroni procedure. Significant GO terms were then shortlisted into representative, non-redundant subsets by focusing on highly specific terms showing corrected p-values lower than 0.01 and by considering the following annotation datasets: Pathways, Molecular Functions, Biological Processes, Cellular Components, Protein Classes, Reactome Pathways.

## Supplementary information

Supplementary Information

Supplementary Tables

## Data Availability

WES data are available at the Molecular Anthropology Lab repository, http://www.bioanthropologybologna.eu.
