# Supplementary material for: A Combined Targeted and Whole Exome Sequencing Approach Identified Novel Candidate Genes Involved in Heritable Pulmonary Arterial Hypertension
Source: Sci Rep. 2019 Jan 24;9:753. doi: 10.1038/s41598-018-37277-0 (PMC6345742; doi:10.1038/s41598-018-37277-0)
Supplement: Supplementary file 1 — Supplementary Information [file 41598_2018_37277_MOESM1_ESM.pdf]

A combined targeted and whole exome sequencing approach identified novel candidate genes involved in heritable pulmonary arterial hypertension

Chiara Barozzi[1#], Margherita Galletti[1#], Luciana Tomasi[1], Sara De Fanti[2], Massimiliano Palazzini[1], Alessandra Manes[1], Marco Sazzini[2*], Nazzareno Galiè[1,3]

[1]Department of Experimental, Diagnostic and Specialty Medicine, University of Bologna, Italy

[2]Department of Biological, Geological and Environmental Sciences, Laboratory of Molecular Anthropology & Centre for Genome Biology, University of Bologna, Italy

[3]National Institute of Biostructures and Biosystems (NIBB), Rome, Italy

[#]These authors contributed equally to this work

*corresponding author (e-mail: marco.sazzini2@unibo.it)

Supplementary Information

Includes Supplementary Figures S1-S3, Legends of Supplementary Tables, Supplementary References.

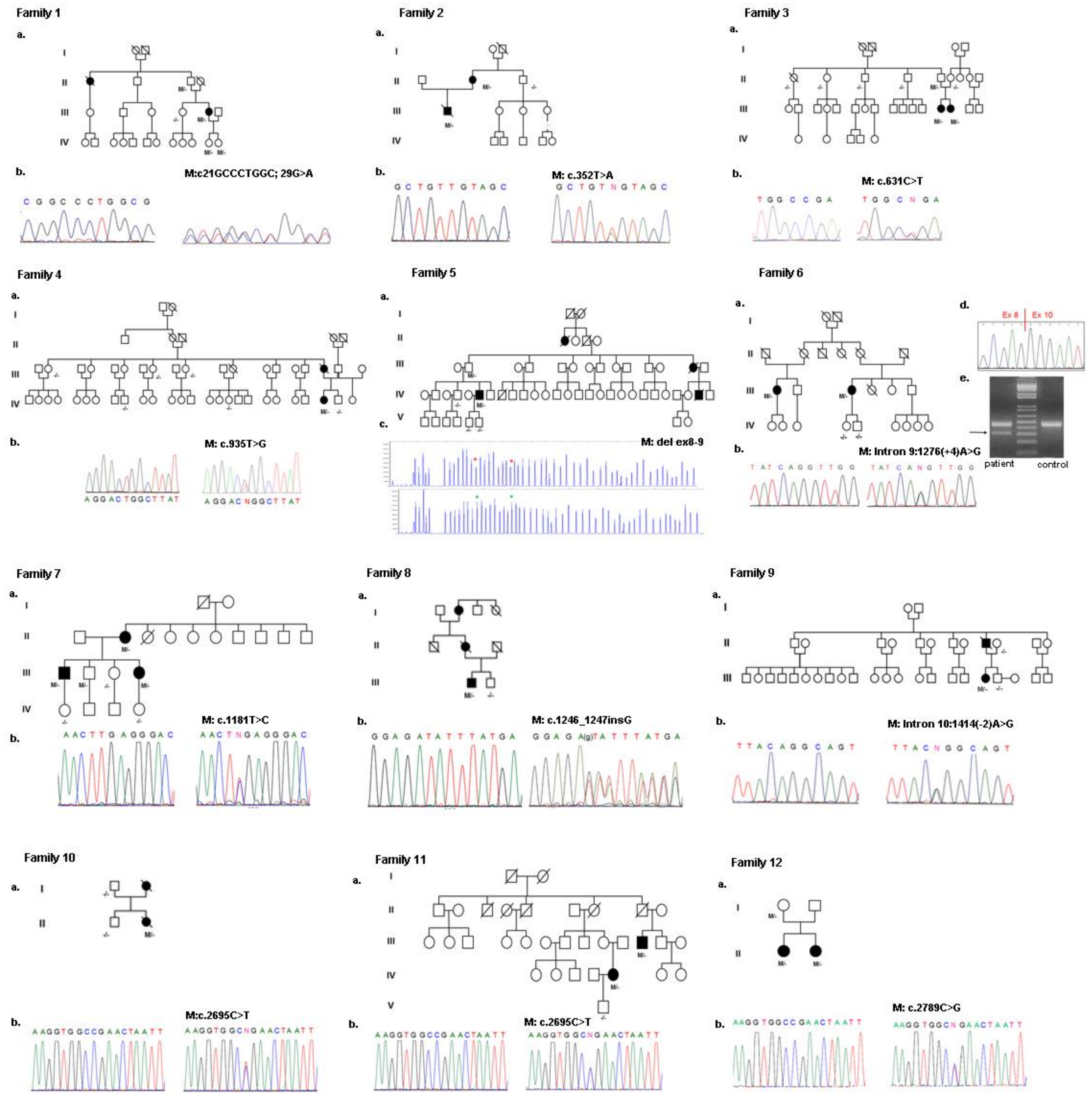

Supplementary Fig. S1. Pedigrees of 12 families with HPAH and details of detected *BMPR2* mutations. a) Pedigrees of 12 HPAH families carrying BMPR2 mutations. Unaffected male □ female ○; affected male ■ female ●; Deceased ⊘ . M, mutated allele; –, wild type allele. **b)** Sanger chromatograms for the identified variants. A, adenine; C, cytosine; G, guanine; T, thymine; N, variant. **c)** MLPA fragment analysis. * Amplicons showing reduced dosage in patients. * Amplicons dosage in control pool. **d)** Sanger chromatograms of *BMPR2* transcript showing exon 9 skipping. **e)** Cropped image of acrylamide gel showing the aberrant transcript due to the occurrence of a splice site mutation (full-length image of the gel is provided as Supplementary Figure S3).

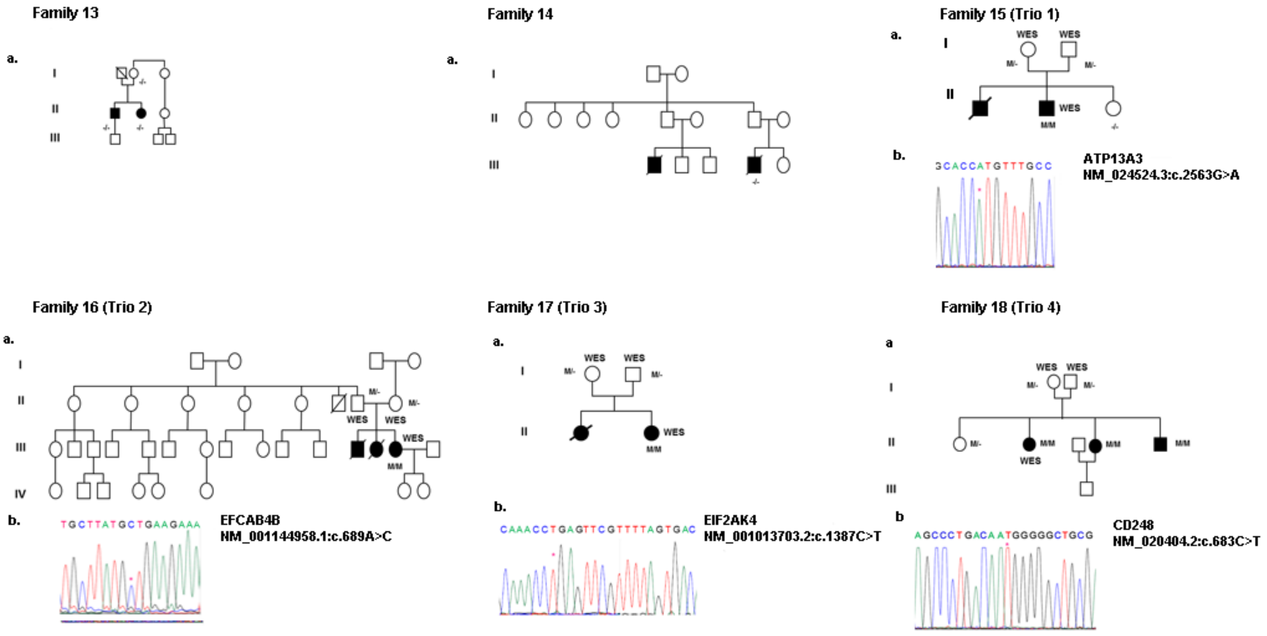

Supplementary Fig. S2. Pedigrees of six families with HPAH and details of related candidate-gene mutations. a) Pedigrees of six HPAH families showing no *BMPR2* mutations. Unaffected male □ female ○ ; affected male ■ female ● ; Deceased ⦰ . M, mutated allele; –, wild type allele. WES, subjects submitted to trio-Whole exome sequencing. **b)** Sanger chromatograms of identified variants. A, adenine; C, cytosine; G, guanine; T, thymine; * variant.

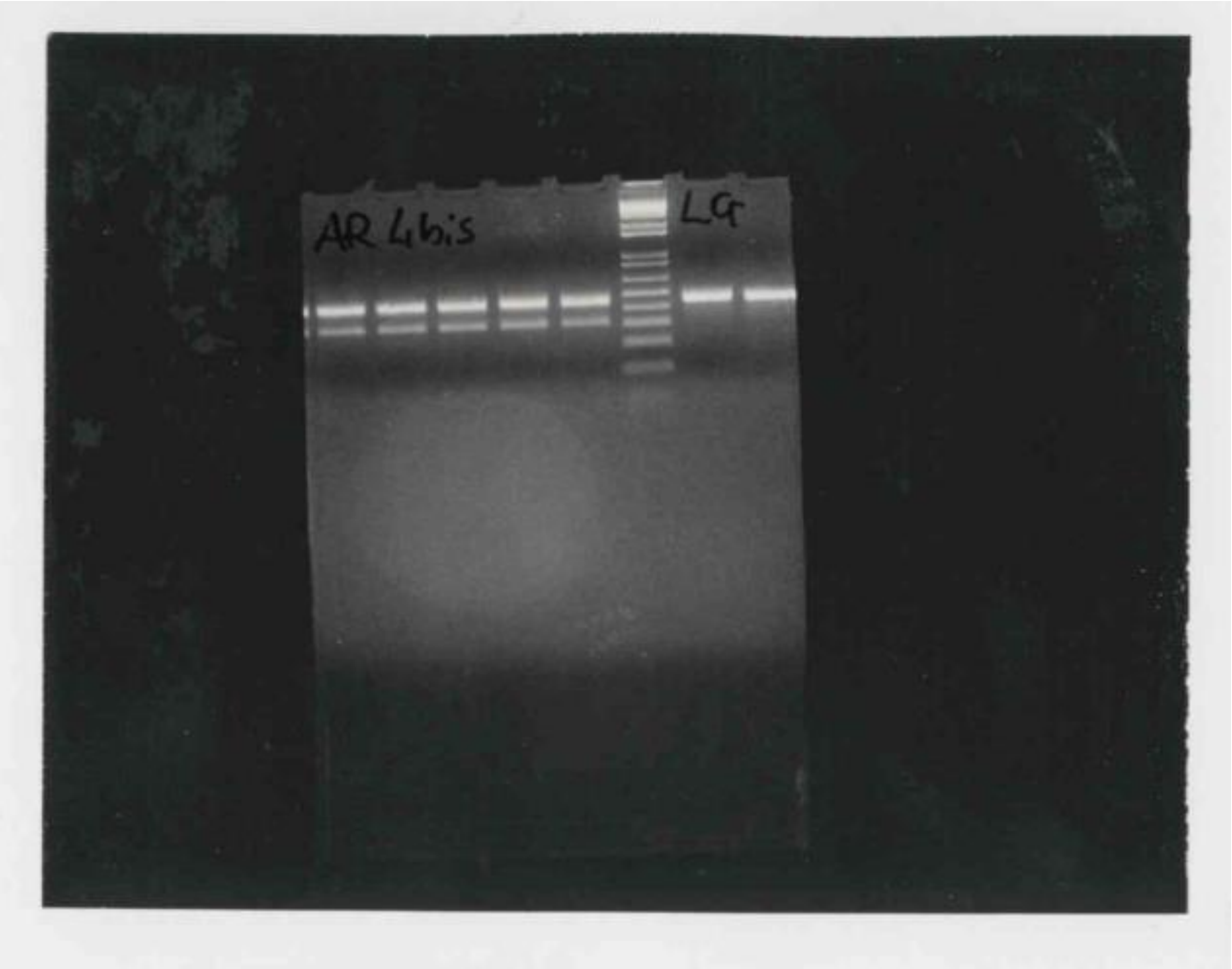

Supplementary Fig. S3. Full-length image of the electrophoresis gel whose detail was reported in Supplementary Fig. S1 (family 6, image e). Individuals showing the aberrant transcript due to the occurrence of a splice site mutation are those presenting two DNA fragments. Control individuals are instead displayed on the right with respect to the DNA size marker and present a single DNA fragment.

Supplementary Tables

Supplementary Tables S1-S4 are included in a separate Excel file.

Supplementary Table S1. Family information and clinical parameters at diagnosis for the 28 HPAH patients included in the study and belonging to 18 families.

Supplementary Table S2. Details of the 26 variants detected via Trio-WES and validated by means of Sanger sequencing.

Supplementary Table S3. Reads depth and ratio of variant reads for genes prioritized via Trio-WES.

Supplementary Table S4. Enrichment analyses performed on genomic regions affected by SNVs and INDELs identified via WES and having passed stringent filtering.

Supplementary References

1. Machado RD, Aldred MA, James V, Harrison RE, Patel B, Schwalbe EC, et al. Mutations of the TGF-beta type II receptor BMPR2 in pulmonary arterial hypertension. *Hum Mutat*. 2006; **27**(2):121-132.
2. Thomson JR, Machado RD, Pauciulo MW, Morgan NV, Humbert M, Elliott GC, et al. Sporadic primary pulmonary hypertension is associated with germline mutations of the gene encoding BMPR-II, a receptor member of the TGF-beta family. *J Med Genet*. 2000; **37**(10):741-745.
3. Aldred MA, Vijayakrishnan J, James V, Soubrier F, Gomez-Sanchez MA, Martensson G, et al. BMPR2 gene rearrangements account for a significant proportion of mutations in familial and idiopathic pulmonary arterial hypertension. *Hum Mutat*. 2006; **27**(2):212-213.
4. Lane KB, Machado RD, Pauciulo MW, Thomson JR, Phillips JA, 3rd, Loyd JE, et al. Heterozygous germline mutations in BMPR2, encoding a TGF-beta receptor, cause familial primary pulmonary hypertension. *Nat Genet*. 2000; **26**(1):81-84.
5. Morisaki H, Nakanishi N, Kyotani S, Takashima A, Tomoike H, Morisaki T. BMPR2 mutations found in Japanese patients with familial and sporadic primary pulmonary hypertension. *Hum Mutat*. 2004; **23**(6):632.
